# White matter is increased in the brains of adults with neurofibromatosis 1

**DOI:** 10.1186/s13023-022-02273-1

**Published:** 2022-03-05

**Authors:** Su Wang, Jan M. Friedman, Per Suppa, Ralph Buchert, Victor-Felix Mautner

**Affiliations:** 1 Department of Medical Genetics, University of British Columbia, Children’s and Women’s Hospital, 4500 Oak Street, Vancouver, BC V6H 3N1 Canada; 2 Department of Nuclear Medicine, Charité - Universitätsmedizin Berlin, Corporate Member of Freie Universität Berlin, Humboldt-Universität zu Berlin and Berlin Institute of Health, Berlin, Germany; 3 Department of Nuclear Medicine, University Hospital Hamburg-Eppendorf, Hamburg, Germany; 4 Department of Neurology, University Hospital Hamburg-Eppendorf, Hamburg, Germany

**Keywords:** Neurofibromatosis 1, Adult brain volumetry, White matter, Neuropsychometric, Age progression

## Abstract

**Background:**

Neurofibromatosis 1 (NF1) is a rare autosomal dominant disease characterized by increased Schwann cell proliferation in peripheral nerves. Several small studies of brain morphology in children with NF1 have found increased total brain volume, total white matter volume and/or corpus callosum area. Some studies (mostly in children with NF1) also attempted to correlate changes in brain morphology and volume with cognitive or behavioural abnormalities, although the findings were inconsistent. We aimed to characterize alterations in brain volumes by three-dimensional (3D) MRI in adults with NF1 in major intracranial sub-regions. We also aimed to assess the effect of age on these volumes and correlated brain white matter and grey matter volumes with neuropsychometric findings in adults with NF1.

**Methods:**

We obtained brain volume measurements using 3D magnetic resonance imaging for 351 adults with NF1 and, as a comparison group, 43 adults with neurofibromatosis 2 (NF2) or Schwannomatosis. We assessed a subset of 19 adults with NF1 for clinical severity of NF1 features and neurological problems and conducted psychometric testing for attention deficiencies and intelligence quotient. We compared brain volumes between NF1 patients and controls and correlated volumetric measurements to clinical and psychometric features in the NF1 patients.

**Results:**

Total brain volume and total and regional white matter volumes were all significantly increased in adults with NF1. Grey matter volume decreased faster with age in adults with NF1 than in controls. Greater total brain volume and white matter volume were correlated with lower attention deficits and higher intelligence quotients in adults with NF1.

**Conclusion:**

Our findings are consistent with the hypothesis that dysregulation of brain myelin production is a cardinal manifestation of NF1 and that these white matter changes may be functionally important in affected adults.

**Supplementary Information:**

The online version contains supplementary material available at 10.1186/s13023-022-02273-1.

## Introduction

Neurofibromatosis 1 (NF1) is an autosomal dominant disease that affects around 1:3000 live births [1]. The disease is characterized by a wide range of features including café-au-lait macules, neurofibromas, central nervous system gliomas, learning disabilities, and attention deficits [2, 3]. Dysregulated growth of myelin-producing Schwann cells due to *NF1* loss of function is a hallmark of the disease [4].

Several studies have examined changes in brain morphology in people with NF1, but most of these studies have small sample sizes and focus on affected children [5–21]. The most consistent findings in individuals with NF1 have been increased total brain volume, total white matter volume and corpus callosum (CC) area [6–21]. Some studies also found increased grey matter volume in children or young adults with NF1 [6, 7, 16]. Other studies have examined grey matter and white matter composition of regional cranial structures, with the most consistent observation being increased corpus callosum area and size [8, 10, 14, 15, 17, 19–21].

We previously examined brain morphology using planar MRI in a large group of adults with NF1 (n = 389) [21]. We found an increase in CC area as well as an increase in brainstem size in comparison to unaffected controls. These structures are mainly composed of white matter, supporting previous findings of white matter enlargement in individuals with NF1.

Individuals with NF1 have cognitive and behavioural abnormalities more frequently than expected. The average intelligence quotient (IQ) of people with NF1 is about one standard deviation lower than the average in the general population [3]. Learning disability occurs in 30% to 70% of individuals with NF1 compared to 8% in the general population, and attention deficit hyperactivity disorder (ADHD) occurs in ~ 40% in individuals with NF1 compared to ~ 10% of the general population [22–25].

Several studies have attempted to correlate changes in brain morphology and volume with cognitive or behavioural abnormalities in individuals with NF1. All of these studies, except one (small sample size using 2D brain morphology measurements) [21], were conducted in affected children. As well, the correlations found were inconsistent across studies, perhaps due to limited sample sizes and numerous variables examined [6–9, 13, 14, 16, 18–20]. Our previous study attempted to correlate CC and brainstem size to the severity of NF1, neurological features, and ADHD, as well as IQ and attentional deficits, but found no associations [21].

In the current study, we compared white matter and grey matter volumes in the whole brain and in major intracranial sub-regions in adults with NF1 and controls. We also assessed the effect of age on these volumes and correlated brain white matter and grey matter volumes with neuropsychometric findings in adults with NF1.

## Methods

### Participants

Between 2003 and 2015, all patients seen in the NF Outpatient Department of the University Hospital Hamburg-Eppendorf in Hamburg, Germany, were offered brain MRI to monitor their intracranial tumour burden. Three-dimensional (3D) magnetization-prepared rapid acquisition with gradient echo (MPRAGE) brain volumetric scans were obtained for 351 adults with NF1 and 43 adults with Neurofibromatosis 2 (NF2) or Schwannomatosis. Adults with NF2 or Schwannomatosis were used as a comparison group as neither condition affects brain volume [26]. If more than one scan was available for a participant, only the most recent scan was included in the analysis.

MRI scans were excluded if classified as outliers for two or more of the 45 volumetric measurements (see *MRI based volumetry*, below). This was done to exclude patients with gliomas or other focal brain lesions. Age and sex were also recorded at the time of the MRI examination. Ninety-nine of the patients with complete brain volumetric scans included in this study are also included in the study of planar MRI scans that we previously reported [21].

A subset of the adults with NF1 and complete brain volumetry measurements (n = 19) also received formal ratings for NF1 severity, neurological severity, and ADHD severity, as well as neuropsychometric assessments for attention deficits and IQ. Fifteen of these 19 patients with NF1 were also included in our previous study of planar brain structural measurements and neuropsychometric measurements [21].

### Brain volumetric measurements

#### MRI preprocessing

MRI brain scans were preprocessed using MATLAB and the Statistical Parametric Mapping toolbox (version SPM12, Wellcome Trust Centre for Neuroimaging, London, UK) (www.fil.ion.ucl.ac.uk/spm). The unified segmentation engine of SPM12 was used to segment the MPRAGE MR images into grey matter (GM), white matter (WM), and cerebrospinal fluid (CSF). Default parameters for segmentation were used except that image data were sampled every 2 mm instead of the default 3 mm [27]. Diffeomorphic anatomical registration through exponentiated Lie algebra (DARTEL) was used for stereotactical normalization of the brain scans into the anatomical standard space of the Montreal Neurological Institute (MNI) [28]. GM and WM component images in native patient space obtained by unified segmentation were resliced to 0.5 mm voxel size prior to the DARTEL registration process in order to avoid aliasing artifacts [29]. DARTEL was used with default parameter settings and the IXI555 templates in MNI space provided by the VBM12 toolbox (http://dbm.neuro.uni-jena.de/vbm). Intensity modulation was applied to the normalized component images to preserve the overall volume.

#### MRI-based volumetry

Volumetric measurements were derived from the modulated tissue component images in MNI space. Total GM, total WM and total CSF volume were obtained by summing all voxel intensities in the corresponding component images and then multiplying the sum by the voxel volume. The total intracranial volume (TIV) was estimated by summing total GM, total WM, and total CSF in a TIV mask predefined in MNI space [30]. Regional GM volume was determined for the following regions of interest (ROI): frontal lobe, parietal lobe, occipital lobe, temporal lobe, cerebellum, inferior frontal gyrus (IFG), insula, hippocampus without subiculum (HC), hippocampus with subiculum (HCS), amygdala, caudate, putamen, and thalamus. Regional WM volume was determined for the following ROIs: frontal lobe, parietal lobe, occipital lobe, temporal lobe, cerebellum, IFG, CC, brainstem, and pons. All ROIs were unilateral and were analysed separately in the left and in the right hemisphere except the ROIs for brainstem, pons, and CC.

The GM or WM volume in a given ROI was obtained by multiplying the corresponding modulated tissue component image (GM or WM) in MNI space with a binary mask of the ROI predefined in MNI space (voxel intensity = 1 for voxels belonging to the ROI, voxel intensity = 0 otherwise), summing all voxels intensities in the resulting masked image, and then multiplying the sum by the voxel volume.

The binary ROI masks were obtained from different brain atlases. The binary masks for frontal, parietal, occipital, temporal lobe, and the cerebellum were derived from the cerebral lobes atlas in MNI space [31]. The binary ROI masks for IFG, insula, caudate nucleus, putamen, and brainstem were derived from the LPBA40 atlas [32]. The ROI mask of the hippocampus without subiculum comprised cornus ammonis and fascia dentate [33]. The ROI mask of the hippocampus with subiculum included the subiculum in addition to cornus ammonis and fascia dentate [33]. The binary ROI masks for amygdala, thalamus and pons were derived from the Automated Anatomic Labeling atlas (amygdala, thalamus) and from the TDlobes atlas (pons), both implemented in the PickAtlas tool of Wake Forest University [34]. The binary CC mask was obtained as the union of binary masks for genu, body and splenium taken from the ICBM-DTI-81 white matter labels atlas [35, 36]. All masks were interpolated to 1.5 mm isotropic resolution to match the resolution of modulated and normalized component images.

The volumetric analyses were performed in batch mode, all MRI scans in a single batch, without visual quality control of spatial normalization and tissue segmentation. Thus, failure of spatial normalization and/or tissue segmentation could not be ruled out. Furthermore, brain lesions that might affect MRI-based volumetry could also not be ruled out. In order to exclude these cases, an MRI scan was considered an ‘outlier’ with respect to a given volumetric measure if the value estimated from this scan was below (lower quartile − 2 × interquartile range) or above (upper quartile + 2 × interquartile range) the expected volume for this entire set of scans. This was tested separately for each volumetric measure. An MRI scan, including all volumetric measurements derived from it, was excluded from all analyses if it was an outlier with respect to two or more of the 45 volumetric measures. 125 (14.5%) 863 MRI scans were identified as outliers and excluded.

### Clinical and neuropsychometric assessments

The clinical assessments [NF1 severity (n = 19), neurological severity (n = 19), ADHD diagnosis (n = 16)], psychometric assessments [IQ (n = 16), and attention comparison scores [ACS] (n = 19)] were obtained in the same manner as previously described [21].

### Statistical analysis

All analyses were conducted in R studio 3.6.0.

#### Demographic analysis

Mean age was compared between adults with NF1 and the comparison group using Student’s T test. Normality and equality of variance were established using the Shapiro–Wilk’s test and Fisher’s F test, respectively. Sex ratios were compared between adults with NF1 and the comparison group using the χ^2^ test.

#### Volumetric analysis

We used the non-parametric Mann–Whitney U test rather than Student’s T test for all volume comparisons because some regional volumes were not normally distributed or the average variance was not the same between hemispheres. Significance values were adjusted using false discovery rate (FDR) to account for 38 volumetric comparisons.

#### Brain volume to age regression analysis

Multiple linear regression was used to determine the effect of sex, age, and NF1 status (as independent variables) on the total brain volume, GM volume, or WM volume, respectively (as the dependent variable). For total brain volume and WM volume, multiple linear regressions were also separately conducted between individuals younger than age 45 years and older than age 45 years. Sex and NF1 status were coded as binary categorical data. The regression models were built using a forward stepwise method, sequentially adding sex, then age, then NF1 status and removing independent variables that were not statistically significant in the subsequent step. The models were compared using ANOVA.

#### Brain volume to neuropsychometric correlation analysis

Select brain volume measurements (total brain volume, GM, WM, white matter CC, white matter brainstem, left and right grey matter frontal lobe, left and right grey matter cerebellum, and left and right grey matter hippocampus without subiculum) were correlated to clinical severity and neuropsychometric measurements using Spearman or Pearson correlation. All brain volume measurements were analysed as continuous data. Clinical assessments (NF1 severity, neurological severity, and ADHD diagnosis) were analysed as ordinal data. Neuropsychometric assessments (IQ and attention deficit [ACS]) were analysed as continuous data. Spearman Rank-Order correlation was conducted for ordinal data and Pearson correlation for continuous data. Significance values were adjusted using FDR to account for all correlation comparisons (n = 16 to 19).

## Results

### Age and sex demographics of study participants

Both age and sex have been shown to affect brain volume in adults [37, 38]. Thus, it is important to determine whether there is a difference in the demographic composition between the NF1 and comparison groups to avoid the effect of sex and age as confounding factors in the analysis. This study included 351 adult participants with NF1 and 43 adult participants with either NF2 or Schwannomatosis (comparison group). Neither age distribution nor sex ratio was different between the two groups (Table 1).

**Table 1:** Participant demographics for adults with NF1 and comparison group (NF2 or Schwannomatosis)

	Adults with NF1	Comparison group	Difference
N	351	43	
Mean age (SD)	37.5 (13.2)	40.0 (14.4)	Not significant
Age range (years)	18.0–79.2	20.1–66.4	
Female:male	205:146 (1.40)	21:22 (0.95)	Not significant

### White matter brain volume is increased in adults with NF1

NF1 is characterized by the dysregulation of myelin formation [4]. In the central nervous system, oligodendrocytes create an insulating myelin sheath around axons [39]. In the brain, myelination is mainly in white matter. To examine the effect of NF1 on myelination in the brain, we investigated the differences in brain volumes (total and regional white and grey matter) between adults with NF1 and individuals without NF1.

**Fig. 1:**
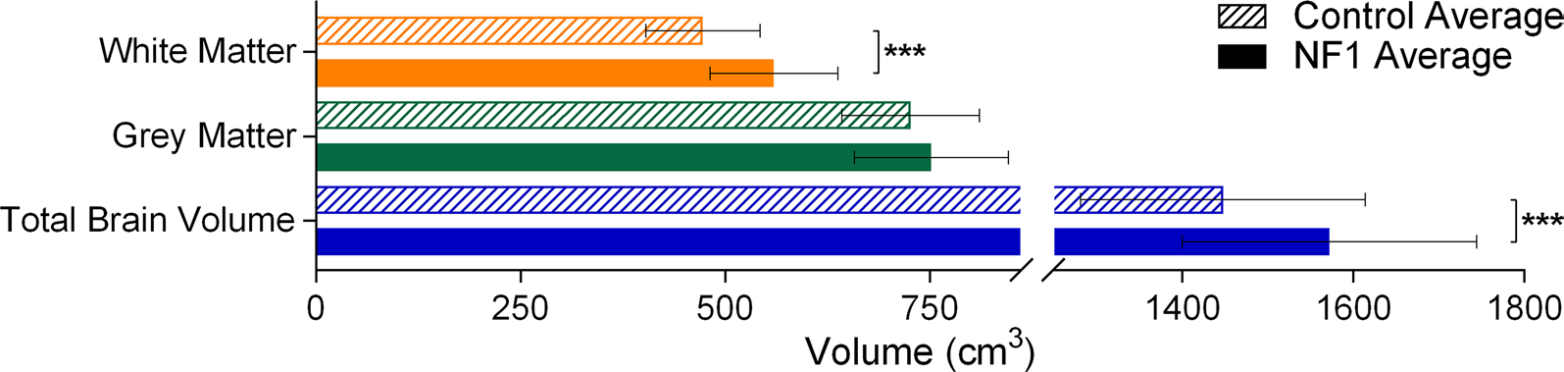
Average total brain volume, grey matter volume, and white matter volume for adults with NF1 and comparison group. Colored bars and outlines indicate different regions measured. Bar fill indicates participant group. Error bars indicate one standard deviation, and asterisks indicate FDR-adjusted statistical significance. ***p < 0.001

**Fig. 2:**
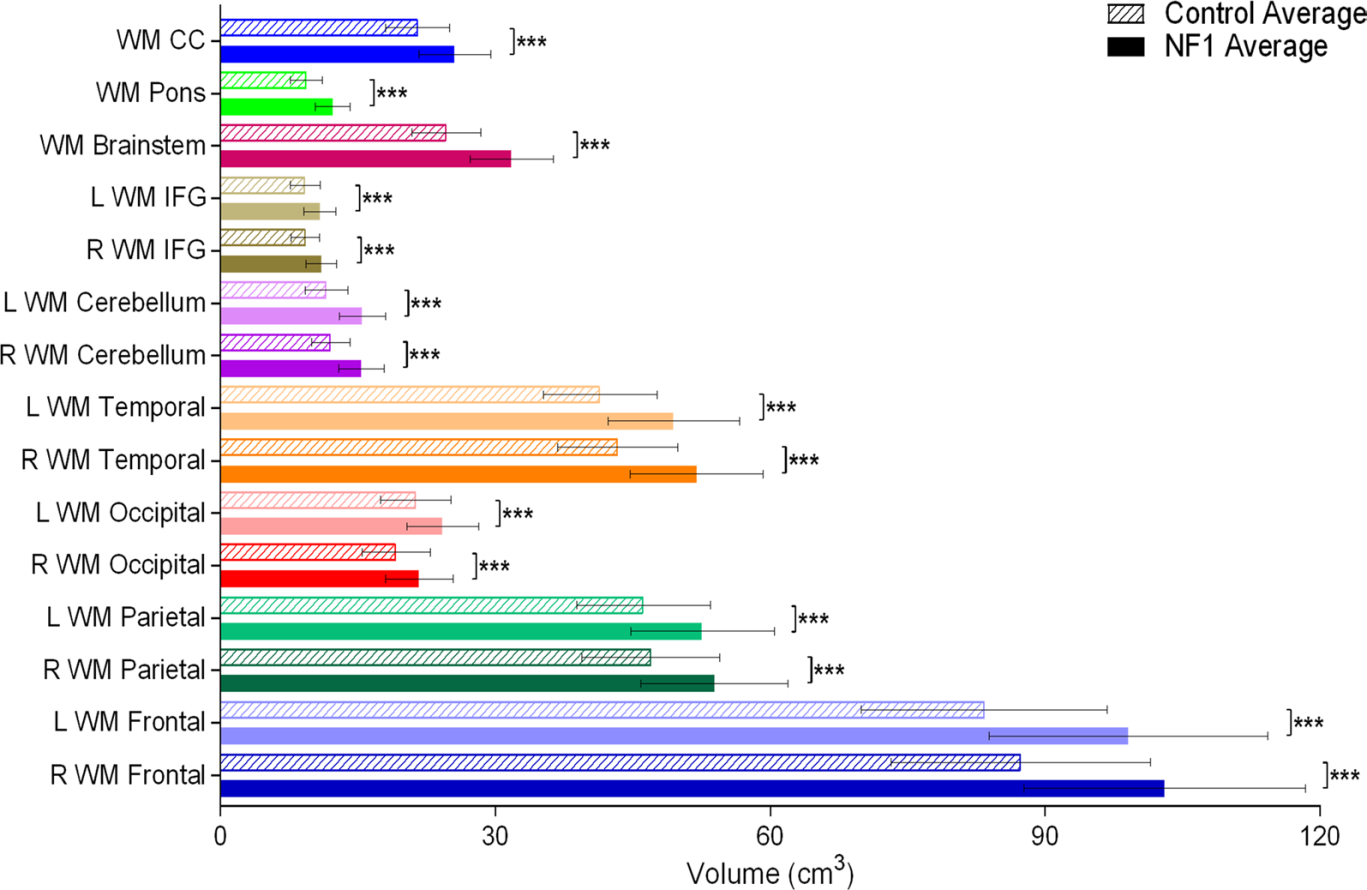
Average regional white matter brain volume for adults with NF1 and comparison group. Colored bars and outlines indicate different regions measured. Bar fill indicates participant group. Error bars indicate one standard deviation, and asterisks indicate FDR-adjusted statistical significance. ***p < 0.001. WM = white matter, R = right, L = left, CC = corpus callosum, IFG = inferior frontal gyrus

**Fig. 3:**
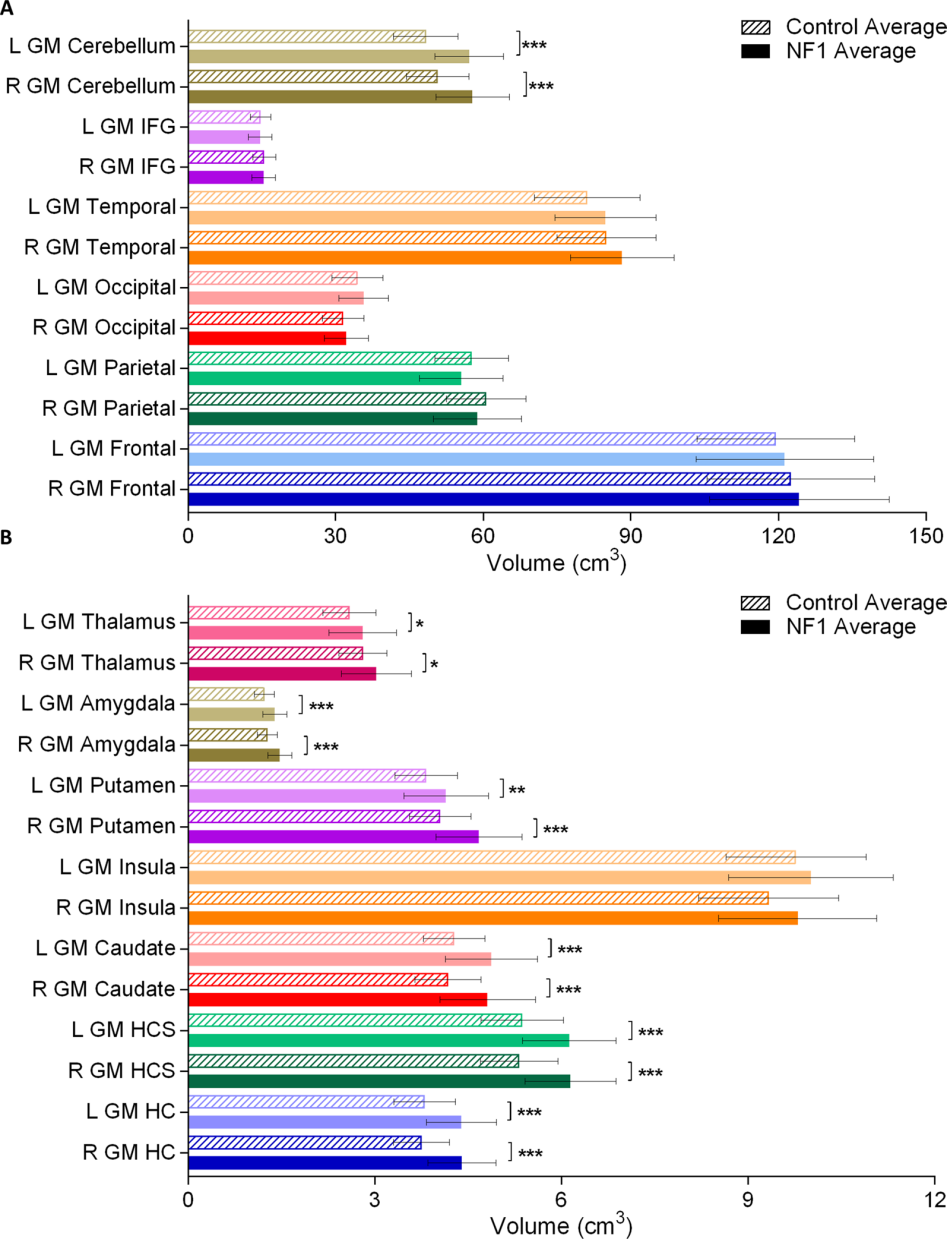
Average major (**A**) and minor (**B**) regional grey matter brain volume for adults with NF1 and comparison group. Colored bars and outlines indicate different regions measured. Bar fill style indicates participant group. Error bars indicate one standard deviation, and asterisks indicate FDR-adjusted statistical significance. *p < 0.05, **p < 0.01, ***p < 0.001. GM = grey matter, R = right, L = left, IFG = inferior frontal gyrus, HC = hippocampus without subiculum, HCS = hippocampus with subiculum

Adults with NF1 have significantly greater total brain volume and total white matter volume than adults in the comparison group (Fig. 1). As well, all the white matter regional measurements (frontal lobe, parietal lobe, IFG, CC, pons, and brainstem) showed a significantly greater volume in adults with NF1 than in those in the comparison group (Fig. 2). This increase in white matter volume was consistent bilaterally. We did not find a difference in total grey matter volume between the NF1 and comparison groups (Fig. 1). However, we found some region-specific changes in grey matter volume. Seven of the 13 regions we measured were significantly larger in adults with NF1 than in the comparison group (Fig. 3). These grey matter volume increases were also consistent bilaterally in all regions.

These results demonstrate that adults with NF1 have uniformly increased white matter volume, and thus more myelin formation, than expected. NF1 also affects grey matter volume, but only in some of the smaller regions of the brain, so that total grey matter volume was not significantly different between adults with NF1 and controls.

### Grey matter volume decreases faster with age in adults with NF1 than in the comparison group

**Fig. 4:**
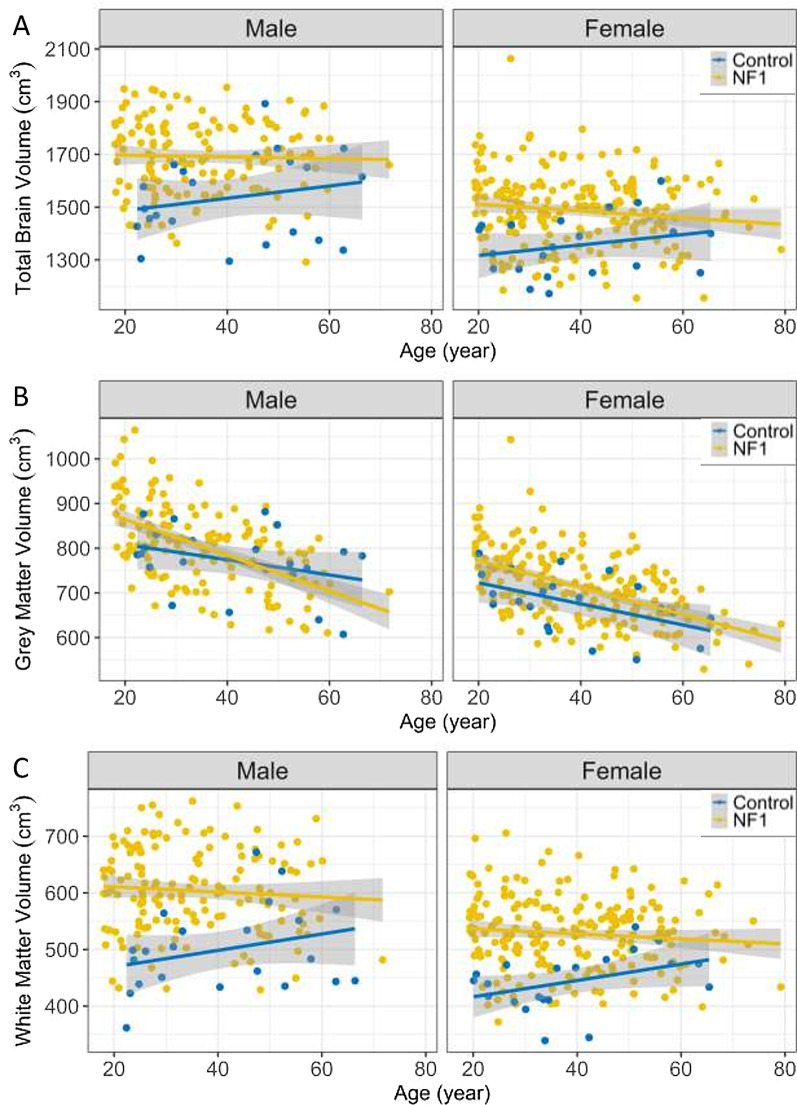
Brain volume measurements by age and sex in adults with NF1 and comparison group. **A** Total brain volume. **B** Total grey matter volume measurements. **C** Total white matter volume measurements. Linear regression lines in panel (**B**) are plotted with 1 standard error range indicated by shading. Each point represents one subject; point and line colour indicate participant group. The regression lines were calculated using individuals of all ages without stratification or segmentation. Age was not a statistically significant predictor of either total brain volume or white matter volume

In the general population, grey matter volume decreases linearly with age, while total brain volume and white matter volume increase with age from early adulthood to around 45 years old, and then decrease [37]. We were interested in whether the rate of reduction in total brain volume, grey matter, and white matter volume over age is different in adults with NF1. We regressed total brain volume, grey matter volume, and white matter volume by age, stratified by sex and NF1 status. We did not find an effect of age on either total brain volume or total white matter volume, either when regressed to the entire age spectrum or separated into younger than or older than age 45 years. (Fig. 4A, C). We found that after adjusting for sex, total grey matter volume decreased with age (p < 0.01) (Fig. 4B, Additional file 1: Table 1) and the adults with NF1 declined significantly faster than adults in the comparison group (p = 0.03) (Fig. 4B, Additional file 1: Table 1).

These results demonstrate that people with NF1 have increased age-related grey matter volume reduction.

### Increased white matter volume is correlated with better psychometric function in adults with NF1

**Fig. 5:**
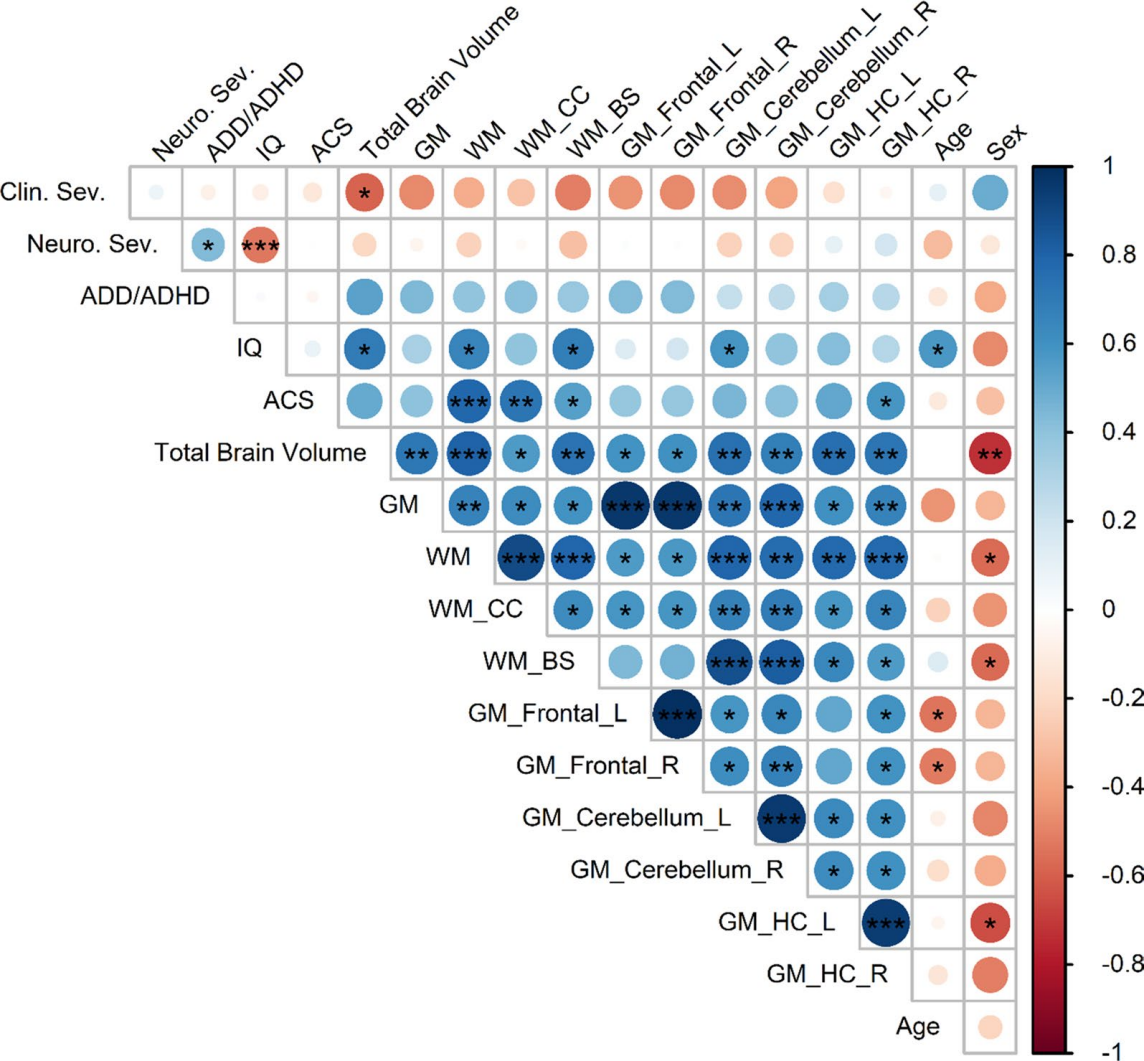
Correlation matrix of brain volume measurements with clinical and neuropsychometric assessments in adults with NF1. The color of each circle represents the degree of correlation from strongly negative (− 1, red) to strongly positive (+ 1, blue), as shown on the vertical scale on the right side of the figure. The size of the circles also indicates the strength of the correlation, with values closer to − 1 or + 1 larger than those that are closer to 0. Asterisks indicate FDR-adjusted statistical significance based on n = 16–19. *p < 0.05, **p < 0.01, ***p < 0.001. Abbreviations: Clin. Sev. = Riccardi clinical NF1 severity score, Neuro. Sev. = clinical neurological severity score, ADD/ADHD = clinical Attention deficit hyperactivity disorder severity score, IQ = intelligence quotient, ACS = Attention Comparison Score, L = left, R = right, GM = grey matter volume, WM = white matter volume, CC = corpus callosum, BS = brainstem, and HC = hippocampus without subiculum

The average IQ is reduced and the frequencies of learning disabilities and attention deficits are increased in people with NF1 in comparison to unaffected individuals [3, 22, 24]. We determined whether the changes in brain volume measurements we found correlate with psychometric functional differences in adults with NF1 (Fig. 5). Due to the limited sample size (N = 16 to 19), we were unable to study the effect of age and sex on these correlations.

We found that greater total brain volume correlated with less clinically severe NF1 (*r*_*s*_ = − 0.58, adjusted p = 0.024) and higher IQ (*r* = 0.69, adjusted p = 0.013). Total grey matter volume was not significantly correlated with any neuropsychometric measurements. However, regional grey matter volumes showed some correlations with psychometric function: greater cerebellum (left) grey matter volume correlated with higher IQ (*r* = 0.59, adjusted p = 0.040) and increased right HC (but not left) grey matter volume correlated with less severe attention deficiency (*r* = 0.59, adjusted p = 0.024). Higher total white matter and regional (CC and brainstem) white matter volume all correlated with better psychometric function (higher IQ and decreased attention deficit).

These results suggest that greater white matter volume might be correlated with better psychometric function in adults with NF1. Neuropsychometric correlations with grey matter volume changes were inconsistent.

## Discussion

The goal of this study was to investigate brain volume, brain composition with respect to grey or white matter, and the correlation of brain volume differences with function in adults with NF1. Our current findings extend our previous study, which showed that adults with NF1 exhibit enlargement of brain structures that are mainly composed of white matter [21], a result similar to that seen in children with NF1.

NF1 is caused by mutations in the *NF1* gene, resulting in reduced ability to produce neurofibromin. This neurofibromin deficiency leads to dysregulated myelin formation in the peripheral and central nervous systems [4]. Previous brain volumetric studies, most of which were done in small groups of children with NF1, have usually found increased total brain volumes and total white matter volumes [6, 7, 9, 11–13, 16–18, 20]. Our previous study, which was the first large-scale MRI investigation of unselected adults with NF1, demonstrated that the corpus callosum and mid-cerebellar peduncle (brain structures that are mainly composed of white matter), are larger than expected in NF1 patients [21]. We correlated the brain structural data to total white matter volume in a small subset of these patients and found a strong correlation between planar MRI measurements of the corpus callosum and brainstem and total white matter volume in adults with NF1 [21]. These findings supported the hypothesis that the enlarged total brain volume seen in individuals with NF1 is due to an overgrowth of myelin that produces an increase in the size of white matter structures.

In the present study, we analysed volumetric MRI data in the whole brain and intracranial sub-regions of adults with NF1 and controls. We found a generalized increase in white matter volume, but we did not observe an increase in total grey matter volume or in in the volume of grey matter in the frontal, parietal, occipital, or temporal lobes. This finding differs from those in previous studies in children and young adults with NF1 that found increased total grey matter volumes [6, 7, 16]. The difference in our findings may reflect a lag in brain development in younger adults with NF1, such as those studied by Karlsgodt et al. [16], with subsequent “catch-up” of the grey matter/white matter ratio in older NF1 patients, as proposed by Moore et al. [7]. Our finding of generalized white matter enlargement in the brains of adults with NF1 is consistent with dysregulated myelin proliferation in individuals with NF1.

Brain composition changes throughout the adult lifespan [37], and people with NF1 have a shorter average lifespan than unaffected people [40]. Thus, we were interested in the changes to white and grey matter volumes by age in adults with NF1. Our finding that age does not affect total brain volume or total white matter volume differs from observations reported in people who do not have NF1, where total brain volume and white matter volume increase from early adulthood to around 45 years old decrease afterwards [37]. Our findings should be interpreted cautiously as they could be influenced by the small size of our comparison group (n = 43) or the paucity of adults with NF1 above 45 years old in our study (n = 18). We note, however, that most previously-reported studies of brain composition changes with age in people who do not have NF1 encompass narrow age ranges, include small sample sizes, and involve participants with various conditions (e.g., psychiatric history or hypertension) that might affect the findings [37]. Interestingly, we were able to show that grey matter volume decreased faster in adults with NF1 than in our comparison group.

In our small study, we found that increased total brain volume and white matter volume were significantly and consistently correlated with better clinical and psychometric function in adults with NF1. We did not find any correlation with total grey matter volume. Some studies in children with NF1 have found correlations between cognitive and behavioural impairment and CC size, total grey matter volume, or total white matter volume [7, 9, 14, 18], although this was not found in other studies [6, 8, 13, 19, 20]. Few studies of brain morphology and neuropsychometric function have been done in adults with NF1, but our previous study found no correlation between corpus callosum size or brain stem size on planar MRI and neuropsychometric function [21]. Fifteen of the nineteen adults with NF1 in the current study were also included in in our prior study, but the findings are inconsistent [21]. This could be due to the use of volume measurements in the current study, which are more sensitive than the planar measurements used in the previous study; the small sample of adults with complete neuropsychometric measurements available in the current study; our inability to study the effects of age and sex as confounding factors on our neuropsychometric correlations; or the possibility that the correlations we observed are spurious. Thus, our morphology to neuropsychometric correlations should be interpreted cautiously.

One of the main limitations of our study is the lack of 3D MRI scans for a comparison group of healthy adults. We used adults with NF2 or Schwannomatosis as a comparison group because neither of these conditions is thought to affect brain volume [26] and MRIs on these patients were available to us. We did not use published brain volumes for healthy adults as those studies included different age ranges, used different 3D MRI scanners, and included volumetric measurements of different regions of the brain.

## Conclusions

Through our brain volumetric MRI analysis of the largest cohort of adults with NF1 studied to date, we showed that white matter volume is increased compared to adults with NF2 or Schwannomatosis. Thus, the increase in white matter volume previously found in children with NF1 persists into adulthood. This supports the hypothesis that NF1 causes a dysregulation of myelin production by Schwann cells in peripheral nerves and by oligodendrocytes in the central nervous system. Characterization of white matter composition and integrity using diffusion weighted imaging and studies of the relationship of the altered myelination to neuropsychometric function in adults with NF1 may help further elucidate the effects of the volumetric changes we observed on neural connectivity and function.

## Supplementary Information

**Additional file 1:** Regression models of age, sex and group on total brain, grey matter, and white matter volume.

## Data Availability

Anonymous data are available for appropriate research purposes through V.F. Mautner, MD.
