# Supplementary material for: White matter is increased in the brains of adults with neurofibromatosis 1
Source: Orphanet J Rare Dis. 2022 Mar 5;17:115. doi: 10.1186/s13023-022-02273-1 (PMC8898512; doi:10.1186/s13023-022-02273-1)
Supplement: Supplementary file 1 — Additional file 1: Regression models of age, sex and group on total brain, grey matter, and white matter volume. [file 13023_2022_2273_MOESM1_ESM.docx]

**Supplemental Table 1: Regression models of age, sex and group on total brain, grey matter, and white matter volume (N=394).** Age was not a statistically significant predictor of either total brain volume or white matter volume.

| REGRESSION OF SEX AND GROUP ON TOTAL BRAIN VOLUME | | | |
| --- | --- | --- | --- |
|  | Coefficient (β) | 95% CI | p Value |
| Intercept | 1546.61 |  | <0.01 |
| Sex (male = 0, female = 1) | -203.07 | [-230.97, -175.17] | <0.01 |
| Group (control = 0, NF1 = 1) | 144.22 | [99.97, 188.47] | <0.01^††^ |
|  | | | |
| REGRESSION OF AGE, SEX, AND GROUP ON GREY MATTER VOLUME | | | |
|  | Coefficient (β) | 95% CI | p Value |
| Intercept | 892.53 |  | <0.01 |
| Age (years) | -3.18 | [-3.71, -2.66] | <0.01 |
| Sex (male = 0, female = 1) | -79.18 | [-93.33, -65.03] | <0.01 |
| Group (control = 0, NF1 = 1) | 24.81 | [2.49, 47.12] | 0.03^†^ |
|  | | | |
| REGRESSION OF SEX AND GROUP ON WHITE MATTER VOLUME | | | |
|  | Coefficient (β) | 95% CI | p Value |
| Intercept | 508.53 |  | <0.01 |
| Sex (male = 0, female = 1) | -73.93 | [-87.61, -60.24] | <0.01 |
| Group (control = 0, NF1 = 1) | 94.11 | [72.40 to 115.81] | <0.01^††^ |
| ^†^ p value obtained by ANOVA comparing regression model estimating effect of age and sex on grey matter volume to regression model estimating effect of age, sex and NF1 status on dependent variable.  ^††^ p value obtained by ANOVA comparing regression model estimating effect of sex on dependent variable (total brain volume or white matter volume) to regression model estimating effect of sex and NF1 status on dependent variable. | | | |
